# The Epidermal Growth Factor Receptor and Its Ligands in Cardiovascular Disease

**DOI:** 10.3390/ijms141020597

**Published:** 2013-10-15

**Authors:** Nader Makki, Kristina W. Thiel, Francis J. Miller

**Affiliations:** 1 Department of Internal Medicine, University of Iowa, Iowa City, IA 52242, USA; E-Mails: nader-makki@uiowa.edu (N.M.); kristina-thiel@uiowa.edu (K.W.T.); 2 Veterans Affairs Medical Center, Iowa City, IA 52242, USA

**Keywords:** EGFR, EGF-like ligands, vascular disease, atherosclerosis, cardiac remodeling

## Abstract

The epidermal growth factor receptor (EGFR) family and its ligands serve as a switchboard for the regulation of multiple cellular processes. While it is clear that EGFR activity is essential for normal cardiac development, its function in the vasculature and its role in cardiovascular disease are only beginning to be elucidated. In the blood vessel, endothelial cells and smooth muscle cells are both a source and a target of EGF-like ligands. Activation of EGFR has been implicated in blood pressure regulation, endothelial dysfunction, neointimal hyperplasia, atherogenesis, and cardiac remodeling. Furthermore, increased circulating EGF-like ligands may mediate accelerated vascular disease associated with chronic inflammation. Although EGFR inhibitors are currently being used clinically for the treatment of cancer, additional studies are necessary to determine whether abrogation of EGFR signaling is a potential strategy for the treatment of cardiovascular disease.

## 1. Introduction

Since the first discovery of the epidermal growth factor receptor (EGFR) in the mid-1970s, over 40,000 papers have described roles in nearly every known cellular process and in numerous disease states. In this review, we focus on the reported activity of EGFR and its ligands in cardiovascular disease.

**Figure 1:**
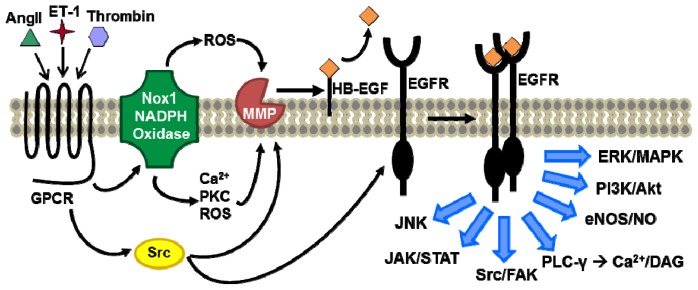
Epidermal growth factor receptor (EGFR) activation either by ligand binding or by transactivation mechanisms results in induction of several downstream signaling pathways.

EGFR is the inaugural member of the ErbB family of receptor tyrosine kinases and consists of an extracellular ligand binding domain, a single α-helical trans-membrane domain, an intracellular tyrosine kinase domain, and a carboxy (*C*)-terminal region that contains autophosphorylation sites. Upon ligand binding, EGFR undergoes a transition from an inactive monomer to an active homodimer or heterodimer with other ErbB family members (ErbB2/HER2/neu, ErbB3, and ErbB4). EGFR dimerization stimulates its intracellular protein tyrosine kinase activity, leading to autophosphorylation of several tyrosine residues in the *C*-terminal domain of the EGFR; consequently, it elicits downstream activation and signaling by several other proteins, including mitogen-activated protein kinases (MAPK), phosphoinositide-3 kinase (PI3K), phospholipase C-γ (PLC-γ), and c-Src [1]. As such, EGFR serves as signaling hub, engaging in cross-talk with multiple pathways (Figure 1). The downstream signaling proteins initiate several signal transduction cascades, principally the extracellular signal-regulated kinase (ERK)/MAPK, PI3K/Akt and jun kinase (JNK) pathways, thereby regulating cell proliferation, survival, differentiation, migration, inflammation, and matrix homeostasis [1]. In addition to the classical pathway of activation, EGFR can be activated through G protein-coupled receptors (GPCR) without direct interaction with GPCR agonists, an event referred to as transactivation [2,3]. EGFR transactivation mediates several downstream signaling cascades such as ERK activation [4,5]. These events are mediated by pathways involving classic second messengers (Ca^2+^, diacylglycerol (DAG)) as well as protein kinases, non-receptors tyrosine kinases, matrix metalloproteinases (MMP), and reactive oxygen species (ROS) [4,6].

In addition to canonical cell-surface EGFR activity, EGFR also localizes to the nucleus via retrograde translocation, where it regulates cell growth via transcriptional activation of cyclin D1 [7,8]. More recently, it has been shown that EGFR is also proteolytically processed, and the intracellular domain fragment is present in both the cytosol and the nucleus [9,10]. Thus, multiple mechanisms exist whereby activated EGFR controls cellular processes.

Ligands of EGFR, which include epidermal growth factor (EGF), transforming growth factor-α (TGF-α), heparin-binding EGF (HB-EGF), amphiregulin (AREG), betacellulin (BTC), and epiregulin (EREG), are synthesized as transmembrane precursors that must be cleaved by MMPs, including the ADAM (a disintegrin and metalloproteinase) family of proteases, to release mature ligands. EGFR ligands are derived from glycoprotein precursors consisting of an extracellular region, a transmembrane domain and a cytoplasmic domain; therefore, proteolytic cleavage can result in release of soluble growth factors [11]. Cleavage occurs between the first and second motifs of the immature ligand precursor, leading to release of the subunit adjacent to the plasma membrane as a mature ligand [12].

While in general the mechanism of cleavage is consistent among the EGFR ligands, some differences have been described. For example, ADAM-mediated liberation of EGF occurs via binding of the ADAM disintegrin domain to EGF and cleavage by its metalloproteinase domain, leading to shedding of mature active EGF [13]. TGF-α is cleaved at two sites within its extracellular domain. The well characterized ADAM17 (also known as tumor necrosis factor-α converting enzyme/TACE) and ADAM10 are capable of cleaving TGF-α at its *N*-terminal site, but the identity of the *C*-terminal protease remains unclear [14]. HB-EGF is primarily cleaved by MMP-3 and MMP-7 [15,16]. Thus, activation of EGFR is complex and multifactorial.

## 2. EGFR in Cardiac Development

EGFR is implicated in proliferation and development of epithelial cells in multiple organs based on evidence that EGFR-deficient mice suffer from abnormalities in the skin, kidney, brain and gastrointestinal tract [17–21]. In these surviving mice and in the *wa-2* mice (mice with a global reduced EGFR kinase activity) the major cardiac defect is valvular, specifically related to the aortic valve. For example, many *wa-2* mice die before weaning. Those surviving to three months develop significantly thicker aortic cusps, culminating in severe aortic stenosis, left ventricular hypertrophy and heart failure [22,23]. Histological examination of hearts from *wa-2* mice revealed extensive calcification and inflammatory changes mimicking those of human aortic stenosis [23]. The cell types mostly affected are epithelial and glial. Roles for the other ErbB family members in cardiac development have also been provided by studies of mice deficient in ErbB2 and ErbB4 [24,25]. Furthermore, a single nucleotide polymorphism of ErbB4 is associated with congenital cardiovascular abnormalities in humans, specifically left ventricular outflow tract defects [26]. In addition, mice deficient in EGF-like ligands, such as HB-EGF, have decreased survival and abnormalities in aortic valve and ventricular development similar to those in *wa*-2 mice and ErbB2-deficient mice [27]. Finally, deficiency of ADAM10 yields similar impairment of heart development, presumably through alterations in EGF-like ligand bioavailability [28]. Taken together, these studies demonstrate essential roles for EGFR family members in normal cardiac development.

## 3. EGFR and Ligands in the Blood Vessel

Multiple cell types within the vascular wall have the potential to express EGFR family members and EGF-like ligands. All four members of the EGFR family of receptors as well as most EGF-like ligands (HB-EGF, TNF-α, BTC, EREG, and AREG) are expressed by vascular smooth muscle cells (VSMCs), and EGFR has also been reported to be expressed on endothelial cells (ECs) [29–31]. In addition, EGFR, HB-EGF, AREG, and EREG are expressed by monocytes and macrophages [30]. Given the contribution of inflammatory cells in vascular diseases such as atherosclerosis, it is important to recognize that these cells provide an additional source for EGF-like ligands. For example, HB-EGF is released by monocytes and acts as a VSMC chemoattractant and mitogen [30].

### 3.1. Vascular Smooth Muscle Cells (VSMCs)

Early evidence for EGFR function in VSMCs was provided by studies in which treatment with EGF was shown to promote bovine VSMC proliferation [32]. More recently, using mice with targeted deletion of EGFR, Schreier and colleagues investigated the importance of EGFR in VSMCs with respect to survival, cellular proliferation and matrix homeostasis [33]. EGFR deletion in these mice was found to promote cellular death, reduce endothelin-1 (ET-1)- or α1-adrenoreceptor-induced ERK1/2 phosphorylation, and disrupt the response to oxidative stress [33]. Indeed, EGFR is well-known to play a key role in the mechanisms underlying VSMC proliferation and migration [34,35]. For example, AREG markedly increases growth of VSMCs [29]. In addition, EGF has been shown to increase cytosolic Ca^2+^ levels and contraction of VSMCs [36].

Transactivation of EGFR by angiotensin II (AngII), ET-1, or bradykinin involves ROS-dependent activation of c-Src, which then activates EGFR and leads to ERK activation and pro-growth signaling [37–39]. ET-1, through acting on ET-1 receptors, has been shown to induce vascular contraction as well as DNA synthesis by transactivating EGFR, which in turn activates the ERK/MAPK pathway [40,41]. VSMC proliferation in response to thrombin also involves EGFR transactivation [42,43].

In addition, Ang II activates EGFR by inducing shedding of pro-HB-EGF by ADAM17; this also leads to activation of ERK as well as PI3K/Akt pathways and VSMC hypertrophy, migration, and proliferation [30,44,45]. This pathway requires second messengers such as Ca^2+^ and ROS and involves phosphorylation by ADAM kinases [30,46]. Similar mechanisms of EGFR transactivation have been proposed for other GPCRs [39,43]. Of note, MMPs and ADAMs contain cysteine residues that are redox sensitive [47]. Recently it has been shown that caveolin-1, the major structural protein of caveolae, negatively regulates expression of ADAM17 in cultured VSMCs, suggesting a potential role for lipid rafts in regulating EGFR activation [48].

Studies from our group have provided a link between EGFR and the NADPH oxidase Nox1, with consequences for proliferation and migration of VSMCs. First, EGFR is transactivated upon thrombin activation of Nox1 [49]. This mechanism involves ROS-dependent MMP shedding of EGF-like ligands, which in turn activate EGFR and downstream PI3K/Akt. Second, migration of VSMCs in response to thrombin also occurs via Nox1 transactivation of EGFR. The EGFR downstream effector ERK induces MMP-9 activity and N-cadherin shedding, thereby promoting VSMC migration [43]. In addition to acting as an upstream regulator of EGFR activation, Nox1 is also downstream of EGFR. For example, extracellular oxidative stress activates EGFR through metalloproteinase-mediated shedding of EGF-like ligands, leading to ERK-dependent activation of the transcription factor ATF-1 and subsequent expression of Nox1 and cell proliferation [50]. We also recently reported that treatment of cultured monkey VSMCs with EGF-like ligands increases expression of Nox1 as well as Nox1-dependent ROS production [31].

### 3.2. Endothelial Cells

As compared to VSMCs, the effect of EGFR activation in ECs has not been as well characterized. Both EGFR transactivation by GPCRs and classical activation by EGF-like ligands participate in EC function. For example, ET-1 activation of cyclooxygenase 2 in microvascular ECs involves transactivation of EGFR [51]. Treatment with HB-EGF induces endothelial nitric oxide synthase (eNOS) expression and nitric oxide (NO) production in human umbilical vein endothelial cells (HUVECs) [52]. Furthermore, HB-EGF induces dilation of arterioles, which can be prevented by inhibition of NOS or by an endothelin B receptor antagonist [53].

## 4. EGFR and Its Ligands in Cardiovascular Disease

**Figure 2:**
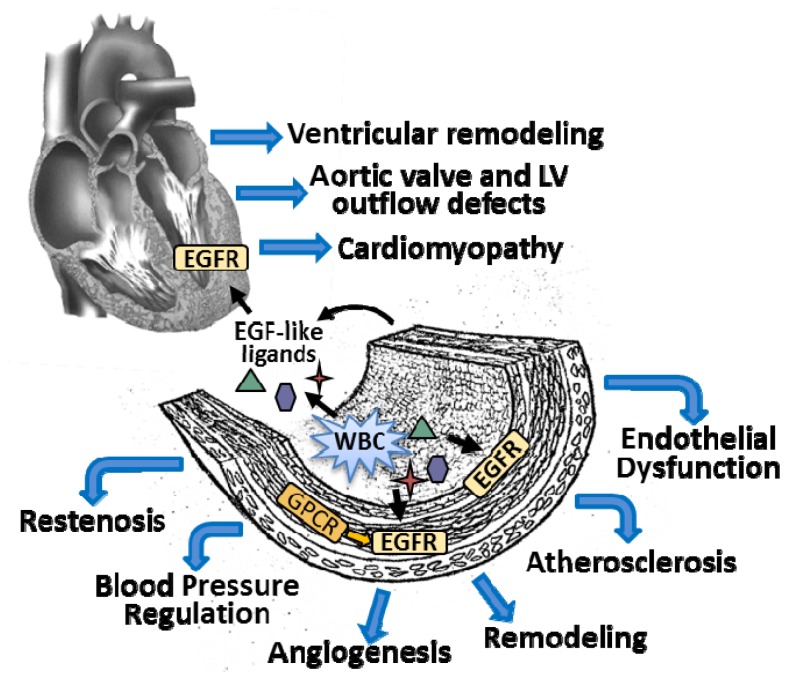
EGFR and its ligands are implicated in multiple vascular disease states. EGFR acts as a switchboard for signaling many cellular processes involved in vascular disease, including cell proliferation, differentiation, survival, migration, matrix homeostasis, and inflammation. EGFR and its ligands are expressed by VSMCs and ECs as well as inflammatory cells.

EGFR activated signaling pathways control cell proliferation, apoptosis, angiogenesis and metastatic spread in several different human cancers. Despite a clear role for EGFR activation in the development of cancer, a disease with many similarities to SMC activation following vascular injury, the role of EGFR in vascular disease is poorly defined. In this section, we describe the current knowledge in the field (Figure 2).

### 4.1. Blood Pressure Regulation

The result of EGFR activation on blood pressure depends on the sum of the effects on endothelial vasodilator release and smooth muscle contraction. As described above, EGF can increase the expression and activation of endothelial NOS and cause vascular dilation [52,53]. In addition, EGF causes VSMC contraction [36] and mediates ET-1 vasoconstriction [41]. Recently, it was shown that mice with smooth muscle targeted deletion of EGFR have reduced diastolic and mean blood pressures, whereas systolic blood pressures are no different [54]. Furthermore, myogenic tone of coronary arterioles is mediated by activation of EGFR and subsequent signaling via the ERK1/2-JAK-STAT3 pathway [55]. ET-1-induced arterial constriction requires MMP-dependent shedding of HB-EGF and subsequent EGFR activation with a rapid increase in intracellular Ca^2+^[56]. Mice deficient in HB-EGF or in EGFR (*wa*-2) demonstrate a markedly blunted increase in blood pressure in response to ET-1 [57]. Furthermore, the EGFR mediates α-adrenergic vasoconstriction in isolated arteries [58]. As compared to normotensive rats, the expression of HB-EGF processing enzymes is increased in arteries from hypertensive rats, and inhibition of MMPs decreases blood pressure [58]. EGFR signaling also is involved in regulation of sodium reabsorption [59]. These observations suggest a role for EGFR in the regulation of vascular tone and blood pressure.

### 4.2. Angiogenesis

Limited studies have examined the role of EGFR and its ligands in angiogenesis in the normal vasculature, whereas a large body of literature in the cancer field documents a link between EGFR and vascular endothelial growth factor (VEGF), a well-known initiator of angiogenesis. For example, EGFR inhibition decreases VEGF expression and tumor vascularization [60]. Multiple mechanisms exist whereby EGFR activation leads to VEGF signaling, including activation of PI3K and MAPK pathways and several transcription factors that regulate VEGF transcription [60]. One might speculate that similar mechanisms exist in the setting of remodeling and neovascularization associated with vascular injury. Indeed, there is evidence for increased EGFR phosphorylation in collateral growth in a model of hind limb ischemia [61]. Moreover, treatment of HUVECs *in vitro* with HB-EGF promotes up regulation of eNOS and release of NO via a PI3K-dependent pathway [52], consistent with a role for HB-EGF in angiogenesis. However, studies using an HB-EGF knockout mouse demonstrate that, while EGFR phosphorylation is decreased in collateral growth in the hind limb ischemia model, there is no difference in arteriogenesis as compared to littermates [61]. Stem cell-based neovascularization following hind limb ischemia can be achieved using stem cells that either express high levels of endogenous EGF or have been stimulated with exogenous EGF [62,63], though the mechanisms are not understood. In cardiac muscle cells, transactivation of EGFR by ET-1 has been shown to induce expression of connective tissue growth factor, which is associated with angiogenesis, in a mechanism that requires activation of ERK and ROS [64]. In a very recent study that was attempting to understand how insulin paradoxically temporally increases retinal edema in diabetes mellitus, it was found that EGFR signaling increases vascular leakage in diabetic mice [65]. Moreover, inhibition of EGFR prevents this effect. In addition, pathologic neovascularization in the retina is abrogated by inhibition of EGFR [66]. Taken together, these data indicate that EGFR contributes to physiologic and pathologic angiogenesis.

### 4.3. Restenosis

Many of the central processes involved in the dysregulated growth of cancer cells are also present in vascular disease, particularly neointimal hyperplasia associated with vascular injury. In patients, the treatment of arterial stenosis by angioplasty or stent implantation is associated with restenosis. Administration of a blocking antibody to EGFR inhibits VSMC proliferation and intimal hyperplasia following balloon injury in animal models of restenosis [67,68]. Nox1 has been implicated in neointimal hyperplasia [69], and data from our group have identified activation of EGFR in the mechanism of increased Nox1 expression in VSMCs [49]. Consistent with these observations, Nox1 expression in aorta correlates with plasma levels of EGF-like ligands [31]. These data suggest a potential role for EGFR in neointimal hyperplasia.

### 4.4. Atherosclerosis

Several observations have suggested a potential role for EGFR signaling in atherogenesis. The expression of the EGFR and its ligands (HB-EGF, BTC, and EREG) are elevated in the vascular lesions of humans with atherosclerosis [30,70]. In addition to vascular cells, infiltrating monocytes and macrophages are another important source of EGF-like ligands in atherosclerosis [30]. Furthermore, plasma levels of HB-EGF are increased in patients with coronary artery disease [71] and correlate with serum cholesterol [72]. We have recently extended these findings by examining non-human primates on an atherogenic diet. Phosphorylation of EGFR in the carotid artery and the vascular and plasma levels of EGF-like ligands are increased in monkeys on an atherogenic diet as compared to a normal diet. Interestingly, these changes normalize within 8 months of returning to a normal diet [31]. The increase in circulating EGF-like ligands likely contributes to the enhanced vascular EGFR activation. This mechanism may also be involved in the association between chronic inflammatory diseases and accelerated atherosclerosis. However, to our knowledge, no studies have examined the effects of increased circulating EGF-like ligands in vascular disease.

Another potential mechanism mediating EGFR signaling is related to the finding that the redox state of plasma is more oxidized in patients with risk factors for atherosclerosis [73]. Our data demonstrate that a more oxidized extracellular redox state causes ADAM/MMP-dependent shedding of EGF-like ligands from VSMCs [50]. Consistent with these data, the expression of several ADAMs is increased in atherosclerotic plaques [74]. Despite these clear associations between expression of EGFR and its ligands in atherosclerosis, no studies have addressed whether modulation of EGFR signaling impacts atherogenesis.

### 4.5. Cardiac Injury and Remodeling

While there is a large body of literature on ErbB family members in the heart, a majority of studies have focused on ErbB2, ErbB4, and neuregulin and not EGFR. For example, dilated cardiomyopathy is associated with inhibition of ErbB2 and ErbB4 signaling, whereas activation of these receptors with neuregulin-1 improves cardiac function [75]. Similar to ErbB2 conditional knockout mice, hearts from HB-EGF null mice exhibit increased chamber size, myofiber hypertrophy, and decreased fractional shortening, signs of severe cardiac dysfunction [27]. It is important to note that the constitutive tyrosine phosphorylation levels of ErbB2 and ErbB4 are reduced in HB-EGF null mice [27]. Decreased survival of cardiomyocytes is one potential cause of the cardiomyopathy in ErbB2 and HB-EGF null mice since partial rescue of ventricular dilation and contractility was observed when the antiapoptotic gene Bcl-xL was overexpressed [76]. ErbB2 transgenic mice display characteristics similar to those found in patients with hypertrophic cardiomyopathy, with susceptibility to adrenergic-induced arrhythmias. Interestingly, the over-expression of ErbB2 induces a concurrent up-regulation of multiple proteins associated with this signaling pathway, including EGFR, ErbB3, ErbB4, PI3K, bcl-2 and multiple protective heat shock proteins. In addition, over expression of ErbB2 causes an anti-apoptotic shift in the ratio of bcl-xS/xL in the heart [77].

ErbB signaling may also have a role in regulating the contractility of the adult heart since ErbB2 and ErbB4 are located in the T-tubule ultrastructural network in cardiomyocytes, the site of excitation-contraction coupling [78]. For example, EGF increases myocardial contractility by elevating cAMP levels in myocytes in a mechanism that requires EGFR tyrosine kinase activity [79,80]. A number of reports have concluded that ERK1/2 and Akt are important for EGFR-mediated cardiomyocyte survival [81–83]. Expression of a dominant negative EGFR mutant in cardiomyocytes results in an increase in the left ventricular mass, consistent with cardiac hypertrophy, and a decrease in fractional shortening that could be normalized by cAMP stimulation [84].

Aldosterone causes inflammation and fibrosis of the heart and remodeling of blood vessels [85]. Interestingly, aldosterone increases the expression of EGFR via the interaction of aldosterone-bound mineralocorticoid receptor with the EGFR promoter [86]. Furthermore, several studies have suggested synergy between aldosterone and AngII via crosstalk that involves EGFR [87]. Inhibition of the EGFR attenuates the AngII-mediated synthesis and release of the extracellular matrix protein fibronectin by cardiac fibroblasts [88]. AngII-dependent cardiac hypertrophy requires cardiac EGFR activation [89], and antisense to EGFR decreases left ventricular hypertrophy in young spontaneously hypertensive rats [90]. However, using a cardiomyocyte-specific overexpression of mutant EGFR, it was recently suggested that, although EGFR contributes to AngII-mediated cardiac remodeling, it may not be necessary for the remodeling effects of aldosterone [91]. In addition, GPCRs have been proposed as potential cardiac preconditioning mimetics. Transactivation of EGFR is required for the protective effect of bradykinin on reducing infarct size in a model of rat ischemia reperfusion [92]. Further investigation is required to resolve the mechanistic contribution of EGFR in cardiac remodeling.

### 4.6. Diabetes Mellitus

Diabetes mellitus increases the risk for multiple cardiovascular complications. Several observations suggest a role for EGFR activation in vascular dysfunction associated with type 1 and type 2 diabetes. EGFR phosphorylation is elevated in the type 2 diabetic mice (db/db) [93]. Inhibition of EGFR signaling normalizes the altered vasoconstrictor and vasodilator responses in streptozocin-induced diabetic rats [94,95] and, in db/db mice, restores eNOS expression, thereby improving both conduit and resistance vessel endothelium-dependent relaxation [93]. Abnormal myogenic tone associated with diabetes is mediated in part via EGFR signaling [96]. In addition, inhibition of EGFR prevents the majority of the changes in gene expression within the rat mesenteric vasculature associated with streptozocin-induced diabetes [97]. These data indicate that activation of EGFR contributes to the vascular dysfunction associated with diabetes.

## 5. Therapeutic Implications

As we have reviewed herein, there is growing evidence for causal roles for EGFR and its ligands in the development and progression of many cardiovascular abnormalities. Mutations in EGFR have been documented in many cancers and impact response to therapy. However, it is important to note that the majority of these mutations are somatic rather than germline mutations and, to date, no mutations in EGFR have been reported that alter its activity in the cardiovascular system.

Based on the role of EGFR and its ligands in cardiovascular disease, EGFR inhibitors should be explored as a therapeutic strategy. Numerous inhibitors of EGFR family members have been developed for clinical applications in solid tumors. To date, three small molecule tyrosine kinase inhibitors (TKIs, EGFR–gefitinib and erlotinib; EGFR/HER2–lapatinib) and four monoclonal antibodies (mAbs, EGFR–cetuximab and panitumumab; HER2–trastuzumab and pertuxumab) have been FDA-approved for use in breast, colorectal, head and neck, non-small cell lung, and pancreatic cancers. The TKIs are ATP analogs that bind in the ATP binding pocket of the tyrosine kinase domain with high affinity, thereby preventing receptor activation. Due to the fairly distinct structure of the tyrosine kinase domain in EGFR, HER2, and HER4, the TKIs targeting these receptors have little effect on catalytic activity of other tyrosine kinase receptors. All FDA-approved TKIs are reversible inhibitors, though irreversible inhibitors are being tested in clinical trials. The mAbs bind to the extracellular domain to antagonize ligand binding and induce receptor internalization without activating the receptor (*i.e.*, cetuximab) or prevent dimerization (*i.e.*, pertuzumab). In addition, some studies of mAbs (*i.e.*, trastuzumab) have demonstrated additional therapeutic benefit due to antibody-dependent cell-mediated cytotoxicity, a process in which binding of the antibody to target cancer cells elicits an anti-tumor immune response [98].

Highlighting the pivotal role for EGFR family members in cardiovascular homeostasis, the EGFR family targeted therapies are associated with adverse cardiac effects, the most notable of which is cardiotoxicity associated with the HER2 mAb trastuzumab [99]. Toxicity ranges from subclinical abnormalities like asymptomatic decline in ejection fraction to more severe events such as congestive heart failure and acute coronary syndrome [100]. While inhibition of EGFR may produce adverse cardiovascular effects, they are generally manageable [99] and do not necessarily preclude consideration of EGFR inhibitors for the treatment of cardiovascular disorders.

## 6. Conclusions

The signaling cascades induced by EGFR activation do not differ significantly among cell types in the vasculature and heart. Despite these similarities, we are only beginning to understand the precise contribution of EGFR and its ligands to normal cardiovascular biology and disease pathogenesis. Whole-animal and conditional knockout mice have shed light on the role of EGFR and its ligands on cardiac development, yet one important issue that remains to be addressed is the redundant and differential functions of EGFR, other ErbB family members, and the various EGFR ligands in cardiovascular function. Future studies that use cell-specific and conditional deletion or overexpression of these receptors and ligands are critical to decipher the full web of influence of EGFR family signaling in cardiovascular biology and disease.
